# Long-Term Prognosis of Different Subtypes of Left Ventricular Noncompaction Cardiomyopathy Patients: A Retrospective Study in China

**DOI:** 10.3390/jcdd10090369

**Published:** 2023-08-28

**Authors:** Shiqi Gao, Shuyuan Zhang, Zeyuan Wang, Ming Wu, Chengying Gu, Ruilian Bai, Meixi Liu, Zhuang Tian, Shuyang Zhang

**Affiliations:** 1 Department of Cardiology, State Key Laboratory of Complex Severe and Rare Diseases, Peking Union Medical College Hospital, Chinese Academy of Medical Science & Peking Union Medical College, Beijing 100730, China; gsq_pumch@163.com (S.G.); 18629952005@163.com (S.Z.); zeyuanw2021@163.com (Z.W.); wu_m17@student.pumc.edu.cn (M.W.); gucy0912@163.com (C.G.); rlbai99@126.com (R.B.); meixiliu_pumc@126.com (M.L.); 2 Medical Research Center, State Key Laboratory of Complex Severe and Rare Diseases, Peking Union Medical College Hospital, Chinese Academy of Medical Science & Peking Union Medical College, Beijing 100730, China

**Keywords:** left ventricular noncompaction (LVNC), transthoracic echocardiography, prognosis, cardiac dilation

## Abstract

Left ventricular noncompaction (LVNC) is a heterogeneous cardiomyopathy that can be classified into different subtypes based on morphologic and functional features. However, the prognosis of the dilated and isolated subtypes of non-pediatric LVNC remains unknown. We retrospectively studied 101 patients with LVNC diagnosed at Peking Union Medical College Hospital from 2006 to 2022 using the Jenni criteria of transthoracic echocardiography. The patients were grouped into those with dilated LVNC (*n* = 64) or isolated LVNC (*n* = 37), and 88 patients (54 with dilated LVNC and 34 with isolated LVNC) were followed up successfully. The primary outcome was major adverse cardiovascular events (a composite of cardiovascular mortality, heart failure, severe ventricular arrhythmia, and systolic embolism). The median follow-up time was 5.24 years. The incidence of major adverse cardiovascular events was 43.2%; patients with dilated LVNC had a higher risk (adjusted hazard ratio, 4.43; 95% confidence interval, 1.24–15.81; *p* = 0.02) than those with isolated LVNC. None of the isolated LVNC patients had cardiovascular deaths or severe ventricular arrhythmias. The risk of systemic embolism was similar between patients with dilated and isolated LVNC. Our findings indicate that transthoracic echocardiography is a useful tool for classifying LVNC into subtypes with distinct clinical outcomes. Dilated LVNC is associated with a poor prognosis, while the isolated subtype is probably a physiological condition.

## 1. Introduction

Left ventricular noncompaction (LVNC) is a rare and unclassified cardiomyopathy characterized by two distinct layers of myocardium with compacted and noncompacted regions, prominent trabeculae formation, and deep intertrabecular recesses [1,2,3]. Echocardiography has revealed that the estimated prevalence of LVNC is 0.02% to 0.14% in the general population [4,5]. The pathogenesis of LVNC is thought to involve arrested cardiac wall maturation at the embryonic stage, leading to impaired compaction of cardiac muscles [6,7]. Nonetheless, a prevalence of up to 8% has been reported among healthy athletes, indicating that LVNC may be an acquired trait due to physiological adaptation of the myocardium [8,9].

The clinical presentation of LVNC shows notable heterogeneity among individuals, with heart failure (HF), arrhythmia, and systemic embolism being the three typical symptom complexes. Furthermore, many patients with LVNC may be asymptomatic and diagnosed only incidentally through cardiac examinations. Several diagnostic criteria for LVNC have been proposed based on either echocardiography or cardiac magnetic resonance imaging [10,11,12,13,14]. The prognosis of LVNC is highly variable among individuals and reported unfavorable prognostic indicators to include advanced age, a reduced left ventricular ejection fraction (LVEF), abnormal electrocardiography (ECG) findings, late gadolinium enhancement in cardiac magnetic resonance (CMR) imaging, mid-basal segmental noncompaction, and right ventricular dysfunction [15,16,17,18]. LVNC is commonly a genetic disease with more than 100 associated genes and pathologic gene variations detectable in approximately 30% to 40% of affected patients. Mutations in sarcomere genes, including *MYH7*, *TTN*, and *MYBPC3*, are the most common abnormalities observed, and these mutations frequently overlap with those seen in dilated cardiomyopathy (DCM) and hypertrophic cardiomyopathy [16,19,20,21]. As such, categorizing LVNC as a distinct cardiomyopathy or a cardiac anatomical phenotype that can occur in both healthy individuals and those with other cardiomyopathies remains a matter of debate, and a standardized approach to its management has not been established.

To effectively identify high-risk patients within the heterogenous LVNC population, a recent proposal suggested subtyping LVNC based on the underlying cardiomyopathy classification. Specifically, LVNC is subclassified into isolated LVNC, dilated LVNC, hypertrophic LVNC, and LVNC with congenital heart disease [22,23]. Isolated LVNC is defined as the presence of isolated LVNC without other structural heart disease and with normal LV structural and functional parameters. The dilated form of LVNC is defined as the presence of cardiomyopathy that concurrently fulfills the diagnostic criteria of LVNC and DCM [24]. Isolated LVNC is generally considered a benign myocardial alteration, with a 5-year incidence of cardiovascular events of <10% [17]. Although a higher risk of mortality and heart transplantation in children with dilated than isolated LVNC has been reported [25], studies comparing the long-term prognosis between LVNC subtypes in non-pediatric patients are limited.

The purpose of our investigation was to compare the long-term outcomes of dilated LVNC and isolated LVNC in non-pediatric patients and evaluate the prognostic value of the LVNC subtyping system. We also examined whether echocardiography is a suitable method for stratifying the heterogeneous LVNC patient population into different subtypes to aid in clinical management.

## 2. Methods

### 2.1. Study Design and Population

**Figure 1:**
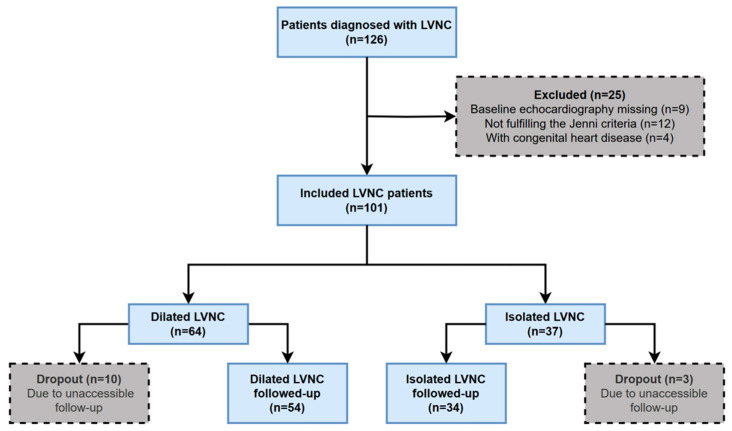
Study flowchart. The diagram describes the inclusion and follow-up of LVNC patients. The diagnosis of LVNC was defined by Jenni’s criteria based on echocardiography. The included patients were divided into dilated LVNC and isolated LVNC according to their echocardiography features. Abbreviations: LVNC, left ventricular noncompaction.

The study workflow is illustrated in Figure 1. We conducted a retrospective review of the electronic medical records of 126 non-pediatric patients admitted to Peking Union Medical College Hospital (PUMCH) from 1 January 2006 to 30 April 2022 to identify patients diagnosed with LVNC. The diagnosis of LVNC was confirmed by experienced researchers using the Jenni criteria, defined as a ratio of the end-systolic noncompacted to compacted layer thickness of ≥2 in any segment of the left ventricle via two-dimensional transthoracic echocardiography (TTE) [11], and for patients whose diagnosis was in doubt on TTE, the Peterson criteria for CMR [15] was further applied for verification of the LVNC diagnosis. We excluded 9 patients with missing TTE records, 12 patients who did not meet the Jenni criteria, and 4 patients with congenital heart disease diagnosed by TTE. The baseline information of the 101 patients included in the study was collected by reviewing the medical records from the first time LVNC was diagnosed at PUMCH. The included patients were stratified into two groups according to the structural and functional parameters of TTE, in which dilated LVNC (*n* = 64) was defined as an LV end-diastolic diameter (LVEDD) of ≥55 mm (male) or ≥50 mm (female) and an LVEF of ≤45% in reference to the definition of DCM [24], while isolated LVNC (*n* = 37) was defined as LVNC with neither LV dilation (not fulfilling the above criteria for dilation) nor other cardiac structural abnormalities. The patients were followed up by telephone or by clinic visit, and the follow-up period was from the LVNC diagnosis at PUMCH to 28 February 2023. During the follow-up period, 10 of 64 patients with dilated LVNC and 3 of 37 patients with isolated LVNC were lost to follow-up because of contact loss or refusal to participate.

### 2.2. Variables

The baseline demographic and clinical characteristics included age, sex, body mass index, baseline New York Heart Association (NYHA) functional class, history of cigarette smoking and alcohol drinking, and complications including hypertension, diabetes mellitus, coronary heart disease, and dyslipidemia. The findings of the ECG and TTE examinations at the time of LVNC diagnosis or those after diagnosis were collected as baseline characteristics, and both examinations were performed and interpreted by experienced specialists. Quantitative baseline TTE parameters included the LVEDD, LV end-systolic diameter, interventricular septum thickness, LV posterior wall thickness, and LVEF. The qualitative TTE characteristics were mitral regurgitation and left atrial (LA) enlargement, which was defined as any LA diameter (anteroposterior, left-to-right, or suprainferior diameter) exceeding the upper reference limits at our institution according to the patient’s age and sex. Laboratory examinations included the lipid profile, cardiac troponin I (cTnI) concentration, brain natriuretic peptide (BNP) concentration, and N-terminal proBNP (NT-proBNP) concentration. Abnormal ECG findings, cTnI elevation, and BNP/NT-proBNP elevation were defined in accordance with international guidelines and recommendations [26,27,28]. Abnormal ECG findings included non-sinus rhythms, QRS abnormalities, and repolarization abnormalities. Elevation of cTnI was defined as a cTnI concentration above the 99th percentile of the upper reference limit. Elevation of BNP or NT-proBNP was defined as a BNP concentration of >35 ng/L or an NT-proBNP concentration of >125 ng/L in patients with sinus rhythm or a BNP concentration of >105 ng/L or an NT-proBNP concentration of >365 ng/L in patients with atrial fibrillation.

### 2.3. Outcomes

A composite of major adverse cardiovascular events (MACE) was the primary endpoint of the study. MACE was defined as the combination of cardiovascular mortality, HF events, including rehospitalization for HF, cardiac resynchronization therapy (CRT) device implantation, and heart transplantation; severe ventricular arrhythmia, including sustained ventricular arrhythmia and ventricular fibrillation; resuscitation from sudden cardiac death and appropriate implantable cardioverter defibrillator discharges; and systemic embolism, defined as embolic stroke or peripheral artery embolism. The secondary endpoints were all-cause mortality and new-onset or worsened arrhythmia in comparison to the baseline status.

### 2.4. Statistical Analysis

Quantitative variables are expressed as the mean ± standard deviation, or median with an interquartile range (25th–75th percentile), while qualitative variables are expressed as the number of cases and percentage. The normality of quantitative variables was examined by the Kolmogorov–Smirnov test. Comparisons between groups were performed using either the Student’s *t*-test or the Mann–Whitney nonparametric test for quantitative variables. For qualitative variables, the chi-squared or Fisher’s exact test was utilized when deemed appropriate. Cumulative incidences over time were demonstrated by the Kaplan–Meier method, and the log-rank test was used to compare the incidence curves. The effect of variables on outcomes was analyzed using univariate and multivariate Cox proportional-hazards regression analyses. Candidate variables for the multivariate Cox regression model were selected by their statistical significance (*p* < 0.05) in the univariate Cox analyses and by clinical possibility. The results of the Cox regression analysis are expressed as a hazard ratio (HR) with a 95% confidence interval (CI). The risk factors associated with dilated LVNC were assessed through multivariate logistic regression analyses. The candidate variables were selected based on demographic and clinical characteristics. Only variables achieving a significance level of *p* < 0.05 between groups or risk factors generally considered for DCM (alcohol consumption) were considered. The results of the logistic regression analyses are expressed as an odds ratio (OR) with a 95% CI. Statistical analysis was conducted using IBM SPSS Statistics 25 (IBM Corp., Armonk, NY, USA). A significance level of *p* < 0.05 was set for all tests to determine statistical significance.

## 3. Results

### 3.1. Baseline Characteristics

The baseline characteristics of the 101 patients with LVNC are shown in Table 1. The age at diagnosis of dilated LVNC and isolated LVNC was 51.2 ± 16.4 and 39.4 ± 13.9 years, respectively; patients with isolated LVNC were generally younger than those with dilated LVNC (*p* < 0.01). Patients with dilated LVNC were more often male (64.1% vs. 43.2%, *p* = 0.04) and presented with worse heart function (NYHA class III or IV) (48.4% vs. 10.8%, *p* < 0.001). There was no difference in body mass index, diagnosis of HF, history of smoking and drinking, hypertension, diabetes mellitus, coronary heart disease, or dyslipidemia between the two subtypes. Patients with dilated LVNC had a significantly larger proportion of abnormal ECG findings than patients with isolated LVNC (75.0% vs. 32.4%, *p* < 0.001). The most frequent ECG abnormalities observed in the dilated subtype were left bundle branch block (21.9% vs. 5.4% in isolated LVNC, *p* = 0.03) and ventricular tachycardia (17.2% vs. 2.7% in isolated LVNC, *p* = 0.05). No statistically significant difference was found in atrial fibrillation, supraventricular tachycardia, or other forms of ECG abnormalities. The baseline quantitative TTE parameters are shown in Figure S1. Patients with dilated LVNC not only had a larger LVEDD [69.0 (62.0–77.0) vs. 53.0 (48.5–56.0) mm, *p* < 0.001] and lower LVEF [27.0 (22.0–34.0) vs. 58.0 (50.0–66.0) mm, *p* < 0.001] according to the stratification criteria, but they also presented with more cases of LA enlargement (95.3% vs. 27.5%, *p* < 0.001) and more severe mitral regurgitation (moderate to severe) (42.2% vs. 2.7%); these could be explained as the consequences of LV dilation. A total of 93.8% of the patients with dilated LVNC, but only 37.8% of those with isolated LVNC, had BNP/NT-proBNP elevation (*p* < 0.001). The pre-existing HF seemed not to be enough to explain the difference in the proportions of BNP/NT-proBNP elevation and NYHA III/IV. And we proposed additional explanations that in patients with dilated LVNC, the increased ventricular end-diastolic pressure and wall tension due to cardiac dilation could stimulate natriuretic peptide secretion, resulting in generally elevated BNP/NT-proBNP levels, and that when hospitalized for HF, the symptoms of dilated LVNC patients (more often NYHA III/IV) were usually more severe than isolated LVNC patients. No difference was observed in the lipid profiles between the two groups of patients. A comparison of the baseline characteristics between the patients who were followed up and those who dropped out revealed no significant differences (Table S1). Multivariate logistic analysis revealed that an age of ≥60 years (OR, 4.19; 95% CI, 1.26–13.93; *p* = 0.02) was the only definite risk factor for dilated LVNC among the demographic and clinical characteristics included in our study (Table S2).

### 3.2. Primary Outcomes

The clinical outcomes of the patients are presented in Table 2. In total, 88 patients diagnosed with LVNC were followed up (54 with dilated LVNC and 34 with isolated LVNC). The median follow-up period was 5.24 (1.55–8.62) years. Thirty-eight patients (43.2%) developed MACE during follow-up, and the incident rate was 9.20 per 100 person-years. There were 8 (9.1%) cardiovascular deaths (incident rate, 1.94 per 100 person-years) and 10 (11.4%) cases of overall mortality (incident rate, 2.42 per 100 person-years). A total of 28 (31.8%) patients developed HF, which included 25 (28.4%) cases of HF rehospitalization, 2 (2.3%) cases of CRT device implantation, and 1 (1.1%) case of heart transplantation. Two (2.3%) cases of severe ventricular tachycardia and nine (10.2%) cases of systemic embolism were also reported.

**Figure 2:**
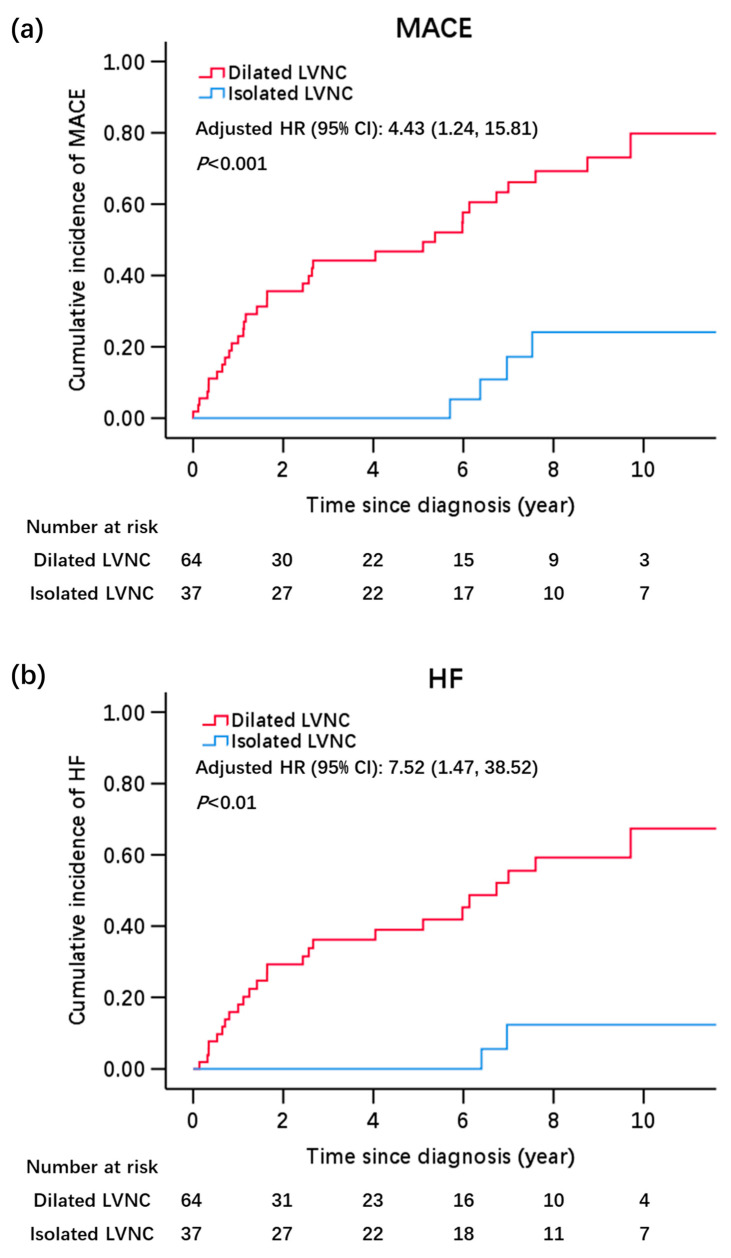
Kaplan-Meier curves for the cumulative incidence of MACE (**a**) and HF (**b**) between the isolated LVNC and dilated LVNC. *p* values were calculated by the log-rank test; adjusted HR and 95% CI were calculated by multivariate Cox proportional-hazards regression analyses. Abbreviations: LVNC, left ventricular noncompaction; MACE, major adverse cardiovascular events; HF, heart failure; HR hazards ratio; CI, confidence interval.

Kaplan–Meier curves for MACE and HF stratified by dilated LVNC and isolated LVNC are plotted in Figure 2. The dilated subtype had a significantly higher incidence of MACE than the isolated subtype (*p* < 0.001). The results of the univariate and multivariate Cox analyses of variables associated with MACE are presented in Table S3. Male sex, age of ≥60 years, smoking, abnormal ECG findings, left bundle branch block, LA enlargement, moderate to severe mitral regurgitation, and BNP/NT-proBNP and cTnI elevation were significantly associated with MACE in the univariate analyses. In the multivariate analyses, three regression models were applied to minimize the confounding effect of other variables (Table 3). Regression models were applied to adjust the risk of dilated LVNC compared with isolated LVNC by basic demographic characteristics (male sex and age of ≥60 years) in Model I, which showed an HR of 6.00 (95% CI, 2.06–17.51; *p* < 0.01). Model II, which was further adjusted by common clinical characteristics associated with cardiovascular events (smoking and NYHA functional class III or IV), showed an HR of 6.93 (95% CI, 2.29–21.00; *p* < 0.01). In the final Model III, which included the results of other examinations (abnormal ECG findings and BNP/NT-proBNP elevation), dilated LVNC showed a significantly increased risk of MACE compared with the isolated subtype, with an HR of 4.43 (95% CI, 1.24–15.81; *p* = 0.02). No other TTE parameters were included in the multivariate regression model; the definitions of dilated and isolated LVNC were already based on a composite pattern of the LVEDD and LVEF, which had strong correlations with other TTE parameters included in our study.

### 3.3. Secondary Outcomes

Kaplan–Meier curves for cardiovascular mortality, systemic embolism, all-cause mortality, and new-onset or worsened arrhythmia between the two LVNC subtypes are plotted in Figure S2 (supplementary materials). Among the eight cardiovascular deaths, none occurred in the isolated LVNC group, and the difference between the Kaplan–Meier curves was significant (*p* = 0.02). Although a multivariate subgroup analysis of cardiovascular mortality could not be performed because of the limited number of events, an increased risk of cardiovascular mortality in patients with dilated LVNC and a relatively favorable long-term prognosis for isolated LVNC could be predicted. Two non-cardiovascular deaths occurred: one patient with dilated LVNC died of pancreatic cancer, and the other with isolated LVNC died of hepatic cancer. After taking these two cases into account, the incidence of overall mortality was also significantly higher in dilated LVNC (*p* = 0.04).

Univariate and multivariate analyses of factors associated with the risk of HF showed results consistent with those of MACE (Table S4). The Kaplan–Meier curves also favored isolated LVNC with a lower incidence of HF (*p* < 0.01). In the multivariate analyses, after stepwise adjustment by age and sex in Model I (HR, 9.88; 95% CI, 2.28–42.68; *p* < 0.01), further adjustment by smoking and NYHA functional class in Model II (HR, 12.76; 95% CI, 2.85–57.13; *p* < 0.01), and final adjustment by ECG findings and BNP/NT-proBNP concentrations in Model III (HR, 7.52; 95% CI, 1.47–38.52; *p* = 0.02), the association of the dilated subtype with increased HF outcomes was still significant, while none of the other variables included in the regression model could partially explain the distinction of HF occurrence between the two groups.

Fourteen (15.9%) patients developed new-onset or worsened arrhythmia (incident rate: 3.39 per 100 person-years), including five (5.7%) cases of atrial fibrillation and four (4.5%) cases of implantable cardioverter defibrillator (CRT) defibrillator implantation, which are the most common types of arrhythmia. Both of these severe ventricular arrhythmia events occurred in the dilated group. Among the 14 cases of new-onset or worsened arrhythmia, 10 (18.5%) occurred in patients with dilated LVNC and 4 (11.8%) occurred in those with isolated LVNC. There was no difference between the two groups (*p* = 0.26), probably because a great portion of arrhythmia events had already occurred before the diagnosis of LVNC and were not reported as clinical outcomes in the follow-up procedure. The number of systemic embolism events was 6 of 54 (11.1%) in the dilated LVNC group and 3 of 34 (8.8%) in the isolated LVNC group. No difference was found in the incidence of systemic embolism using the Kaplan–Meier method (*p* = 0.53). Because of the limited number of events, multivariate analyses of severe ventricular arrhythmia and systemic embolism could not be performed.

## 4. Discussion

To the best of our knowledge, this study is the first to compare the long-term prognosis between non-pediatric patients with dilated LVNC and isolated LVNC. In our retrospective cohort study, 101 patients were included at baseline, and 88 patients with LVNC were followed up for a median of 5.24 years. The similarity of the baseline characteristics between the follow-up and drop-out groups precluded the potential bias introduced by loss to follow-up in our study. The incidence of MACE was 43.2%, and the overall incidence rate of cardiovascular mortality was 1.94 per 100 person-years, which is consistent with the findings of previous studies on LVNC [15,16,29].

This study identifies two subtypes of LVNC based on echocardiographic features and provides evidence that the risk of cardiovascular events significantly differs between the two subtypes. The adjusted HR of MACE was more than 4-fold higher in dilated LVNC, while the effects of the other variables, including age, sex, ECG abnormalities, and BNP/NT-proBNP elevations, were not significant in the multivariate regression model. Furthermore, in clinical practice, medication against ventricular remodeling is more inclined to be initiated in dilated LVNC patients with existing left ventricular dilation and reduced systolic function, whereas the strategy in isolated LVNC patients with no obvious clinical symptoms is often to observe rather than initiate drug therapy. As we did not include the medication information in our analysis, the risk of dilated LVNC might even be underestimated.

The high cardiovascular risk in patients with dilated LVNC was in line with our expectations because a decreased LVEF is commonly regarded as a prognostic factor for adverse cardiovascular events as well as the key indicator for the classification of HF [26]. By contrast, the risk of MACE in patients with dilated LVNC (HR, 4.43) was higher than that of patients with decreased LVEF (HR, 1.74–2.49) in a previous cohort study [16]. A meta-analysis involving 1910 patients from 20 cohorts revealed that an increased LVEDD was an independent risk factor for a poor prognosis apart from the LVEF [17], indicating that using the dilated LVNC subtype defined by both the LVEF and LVEDD is a more comprehensive and discriminable way to perform risk stratification among patients with LVNC than using the LVEF alone. Additionally, MACE occurrences in patients with dilated LVNC are generally more severe. By contrast, no cardiovascular death occurred in patients with isolated LVNC during the long-term follow-up in our study, which aligns with another study that showed no cardiovascular death among 42 patients with LVNC who had an LVEF of ≥45% during a follow-up period of >5 years [30].

Although the occurrence of MACE, cardiovascular mortality, and HF was distinctly different between dilated and isolated LVNC, the occurrence of systemic embolism was similar. The similar baseline incidence of atrial fibrillation, as well as the similar proportion of patients under antiplatelet or anticoagulant therapy, might partially explain why no difference was observed in embolic events. However, the actual stroke risk between the groups was difficult to evaluate because the limited number of embolic events did not allow further adjustment of other variables.

Of the 34 patients with isolated LVNC, 20 underwent at least one additional TTE examination during clinical follow-up. Only 1 of the 20 (5%) isolated LVNC patients with follow-up echocardiography records progressed into the dilated type within 7 years after LVNC diagnosis (whose LVEF decreased to 45%, the boundary value), while others did not develop cardiac dilation regardless of endpoint events. This suggests that isolated LVNC and dilated LVNC are two distinct subtypes rather than two stages in the natural course of the disease. Therefore, we could conclude that dilated LVNC and isolated LVNC are two clinical entities with different long-term clinical prognoses and that TTE is a simple and effective method to distinguish patients with dilated LVNC who have a high risk of MACE. When a patient’s echocardiography suggests myocardial noncompaction accompanied by LVEF < 45% as well as left ventricular dilation, even though the patient’s current symptoms might not be severe, his future cardiovascular risk is high, and more concerns about drug or implantable device therapy should be addressed by the physician. The results of our study also support the opinion that LVNC is more likely to be a cardiac anatomical variation because a low incidence of cardiovascular events was observed in the isolated LVNC group, and excessive examinations and medications might not be necessary for these patients.

The main limitation of this study is that LVNC is a rare cardiomyopathy. This hindered the sample size and limited the number of variables in the regression model, preventing a subgroup survival analysis. Moreover, the retrospective nature of the study had inevitable limitations, such as patient dropout; however, we were able to control missing data in our long-term study to some extent (12.9%), and the demographics of the patients were similar between the two groups. Another limitation is that we only considered the natural progression of the disease. Any interventions through medications such as anti-HF or anticoagulant therapy (which has improved over the last decade) were not considered and could have had an influence on the results. Finally, our research concentrated only on the correlation between phenotype and clinical consequences. More genetic assessments are required to compare genetic variations between different subtypes of LVNC to determine whether the heterogeneity of LVNC has a genetic foundation.

## 5. Conclusions

Non-pediatric patients with LVNC can be classified into subtypes with different prognoses based on TTE structural and functional parameters. Dilated LVNC is significantly associated with an increased risk of MACE, while isolated LVNC has a favorable prognosis, with no cardiovascular deaths observed in long-term follow-up.

## Figures and Tables

**Table 1:** Baseline characteristics of patients with dilated LVNC and isolated LVNC.

Baseline Characteristics	Dilated LVNC (*n* = 64)	Isolated LVNC (*n* = 37)	*p* Value
Demographic and clinical characteristics			
Age of diagnosis, years	51.2 ± 16.4	39.4 ± 13.9	<0.01
Age ≥ 60 y	22 (34.4)	4 (10.8)	<0.01
Male	41 (64.1)	16 (43.2)	0.04
Body mass index, kg/m^2^	22.8 ± 3.5	23.9 ± 3.5	0.19
Diagnosis of HF	41 (64.1)	18 (48.6)	0.13
NYHA cardiac function class			
I or II	33 (51.6)	33 (89.2)	<0.001
III or IV	31 (48.4)	4 (10.8)	
Hypertension	16 (25.0)	13 (35.1)	0.28
Diabetes mellitus	8 (12.5)	4 (10.8)	>0.99
Dyslipidemia	25 (39.1)	6 (16.2)	0.02
Coronary heart disease	8 (12.5)	1 (2.7)	0.15
Cigarette consumption	29 (45.3)	11 (29.7)	0.12
Alcohol intake	29 (45.3)	12 (32.4)	0.20
Electrocardiography features			
Abnormal ECG	48 (75.0)	12 (32.4)	<0.001
LBBB	14 (21.9)	2 (5.4)	0.03
AVB	2 (3.1)	1 (2.7)	>0.99
Atrial fibrillation	9 (14.1)	2 (5.4)	0.18
Atrial flutter	1 (1.6)	0 (0.0)	>0.99
Supraventricular tachycardia	3 (4.7)	3 (8.1)	0.67
Ventricular tachycardia	11 (17.2)	1 (2.7)	0.05
Premature ventricular contraction	12 (18.8)	4 (10.8)	0.29
Bradycardia	1 (1.6)	1 (2.7)	>0.99
ST-T changes	10 (15.6)	3 (8.1)	0.36
Echocardiography features			
LVEDD, mm	69.0 (62.0–77.0)	53.0 (48.5–56.0)	<0.001
LVESD, mm	61.0 (52.0–67.0)	35.0 (31.0–41.5)	<0.001
LVEF, %	27.0 (22.0–34.0)	58.0 (50.0–66.0)	<0.001
IVS, mm	7.0 (6.3–8.8)	8.0 (7.0–9.0)	0.10
LVPW, mm	8.0 (7.0–9.0)	8.0 (7.0–10.0)	0.31
LA enlargement	61 (95.3)	14 (27.5)	<0.001
MV regurgitation			
None to mild	37 (57.8)	36 (97.3)	<0.001
Moderate to severe	27 (42.2)	1 (2.7)	
Laboratory examinations			
Lipids profile, mmol/L			
Total cholesterol	3.89 (3.22–4.66)	4.55 (3.29–4.99)	0.20
Triglycerides	1.10 (0.73–1.54)	1.39 (0.75–2.11)	0.25
HDL-C	0.99 (0.78–1.14)	1.01 (0.82–1.20)	0.85
LDL-C	2.43 (1.90–3.34)	2.43 (1.83–2.80)	0.46
cTnI elevation ^§^	19 (36.5)	4 (16.7)	0.08
BNP or NT-proBNP elevation ^※^	60 (93.8)	14 (37.8)	<0.001
Medications, No. (%)			
ACEI/ARB/ARNI	25 (39.1)	13 (35.1)	0.70
Beta blocker	26 (40.6)	13 (35.1)	0.59
MRA	16 (25.0)	6 (16.2)	0.30
Diuretics	11 (17.2)	4 (10.8)	0.39
Antiarrhythmic drugs	2 (3.1)	0 (0.0)	0.53
Antiplatelet drugs	5 (7.8)	2 (5.4)	>0.99
Anticoagulant drugs	7 (10.9)	2 (5.4)	0.48

Abbreviations: LVNC, left ventricular noncompaction; HF, heart failure; NYHA, New York Heart Association; ECG, electrocardiography; LBBB, left bundle branch block; AVS, atrioventricular block; LVEDD, left ventricular end-diastolic diameter; LVESD, left ventricular end-systolic diameter; LVEF, left ventricular ejection fraction; IVS, interventricular septum; LVPW, left ventricular posterior wall; LA, left atria; MV, mitral valve; HDL-C, high-density lipoprotein cholesterol; LDL-C, low-density lipoprotein cholesterol; cTnI, cardiac troponin I; BNP, brain natriuretic peptide; NT-proBNP, N-terminal pro-brain natriuretic peptide; ACEI, angiotensin-converting enzyme inhibitor; ARB, angiotensin receptor blocker; ARNI, angiotensin receptor neprilysin inhibitor; MRA, aldosterone receptor antagonist. Values were given as mean ± SD, number (%), or median (interquartile range). *p* values were calculated by the Student *t*-test or Mann–Whitney nonparametric test for quantitative variables and Chi-square test or Fisher’s precise test for qualitative variables, when appropriate. ^§^ Elevation in cTnI was defined as baseline cTnI above the 99th percentile upper reference limit. ^※^ Elevation in BNP or NT-proBNP was defined as BNP > 35 ng/L or NT-proBNP > 125 ng/L in patients with sinus rhythm, or BNP > 105 ng/L or NT-proBNP > 365 ng/L in patients with atrial fibrillation.

**Table 2:** Incidence of clinical outcomes in LVNC patients.

Clinical Outcomes	Overall (*n* = 88)	Dilated LVNC (*n* = 54)	Isolated LVNC (*n* = 34)	Overall Incidence Rate (Events Per 100 Person-Years)
Follow-up time, year	5.24 (1.55–8.62)	5.11 (1.34–8.34)	6.79 (2.46–9.66)	-
Primary endpoint				
MACE	38 (43.2)	34 (63.0)	4 (11.8)	9.20
Cardiovascular mortality	8 (9.1)	8 (14.8)	0 (0.0)	1.94
HF	28 (31.8)	26 (48.1)	2 (5.9)	6.78
Rehospitalization for HF	25 (28.4)	23 (42.6)	2 (5.9)	-
CRT implantation	2 (2.3)	2 (3.7)	0 (0.0)	-
Heart transplantation	1 (1.1)	1 (1.9)	0 (0.0)	-
Severe ventricular arrhythmia	2 (2.3)	2 (3.7)	0 (0.0)	0.48
Ventricular fibrillation	1 (1.1)	1 (1.9)	0 (0.0)	-
Appropriate ICD discharge	1 (1.1)	1 (1.9)	0 (0.0)	-
Systemic embolism	9 (10.2)	6 (11.1)	3 (8.8)	2.18
Embolic stroke	6 (6.8)	4 (7.4)	2 (5.9)	-
Peripheral artery embolism	4 (4.5)	3 (5.6)	1 (2.9)	-
Secondary endpoint				
All-cause mortality	10 (11.4)	9 (16.7)	1 (2.9)	2.42
New onset or worsened arrhythmia	14 (15.9)	10 (18.5)	4 (11.8)	3.39
Ventricular fibrillation	1 (1.1)	1 (1.9)	0 (0.0)	-
Appropriate ICD discharge	1 (1.1)	1 (1.9)	0 (0.0)	-
Paroxysmal ventricular tachycardia	1 (1.1)	1 (1.9)	0 (0.0)	-
ICD implantation	4 (4.5)	4 (7.4)	0 (0.0)	-
Pacemaker implantation	1 (1.1)	1 (1.9)	0 (0.0)	-
Atrial fibrillation	5 (5.7)	3 (5.6)	2 (5.9)	-
Supraventricular tachycardia	2 (2.3)	2 (3.7)	0 (0.0)	-
Atrioventricular block	2 (2.3)	0 (0.0)	2 (5.9)	-

Abbreviations: LVNC, left ventricular noncompaction; MACE, major adverse cardiovascular events; HF, heart failure; CRT, cardiac resynchronization therapy; ICD, implantable cardioverter defibrillator. Values were given as a number (%) or median (interquartile range).

**Table 3:** Comparison of incidence of MACE and HF in patients with dilated LVNC and with isolated LVNC.

Outcomes	Isolated LVNC (*n* = 34)	Dilated LVNC (*n* = 54)	*p* Value
MACE			
HR (95% CI), Model I *	1.0	6.00 (2.06, 17.51)	<0.01
HR (95% CI), Model II ^†^	1.0	6.93 (2.29, 21.00)	<0.01
HR (95% CI), Model III ^§^	1.0	4.43 (1.24, 15.81)	0.02
Heart failure			
HR (95% CI), Model I *	1.0	9.88 (2.28, 42.86)	<0.01
HR (95% CI), Model II ^†^	1.0	12.76 (2.85, 57.13)	<0.01
HR (95% CI), Model III ^§^	1.0	7.52 (1.47, 38.52)	0.02

Abbreviations: LVNC, left ventricular noncompaction; MACE, major adverse cardiovascular events; HR, hazards ratio; CI, confidence interval. HR (95% CI) and *p* values were calculated by using the Cox proportional-hazards regression model. * Model I adjusted for age and sex. ^†^ Model II further adjusted for cigarette consumption and NYHA functional class. ^§^ Model III further adjusted for cigarette consumption, NYHA functional class, abnormal electrocardiography, and BNP/NT-proBNP elevation.

## Data Availability

All data relevant to the study are included in the article.

## Supplementary Materials

The following are available online at https://www.mdpi.com/article/10.3390/jcdd10090369/s1, Figure S1: Distribution of echocardiography structural and functional parameters in dilated LVNC and isolated LVNC patients, Figure S2: Kaplan-Meier curves for the cumulative incidence of cardiovascular death, systemic embolism, all-cause death, and new onset or worsened arrhythmia stratified by isolated LVNC and dilated LVNC, Table S1: Comparison of baseline characteristics between followed-up and dropped-out patients, Table S2: Multivariate analysis of risk factors associated with dilated LVNC, Table S3: Univariate and multivariate analysis of variables associated with MACE among patients with LVNC, Table S4: Univariate and multivariate analysis of variables associated with heart failure among patients with LVNC.
