# Supplementary material for: Long-Term Prognosis of Different Subtypes of Left Ventricular Noncompaction Cardiomyopathy Patients: A Retrospective Study in China
Source: J Cardiovasc Dev Dis. 2023 Aug 28;10(9):369. doi: 10.3390/jcdd10090369 (PMC10531838; doi:10.3390/jcdd10090369)
Supplement: Supplementary file 1 [file jcdd-10-00369-s001.zip › jcdd-2512025-supplementary.pdf]

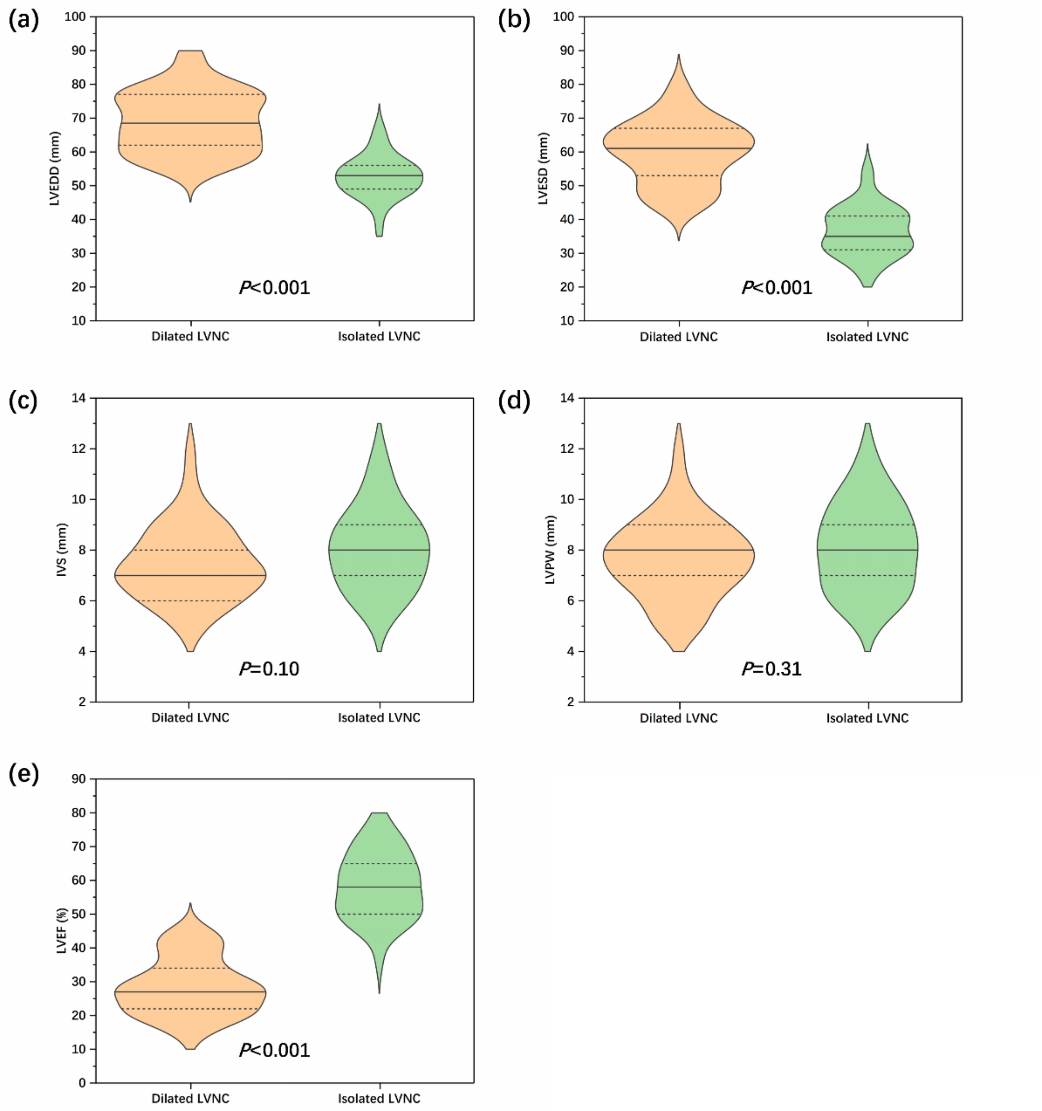

Figure S1. Distribution of echocardiography structural and functional parameters in dilated LVNC and isolated LVNC patients. Abbreviations: LVNC, left ventricular noncompaction; LVEDD, left ventricular end-diastolic diameter; LVESD, left ventricular end-systolic diameter; LVEF, left ventricular ejection fraction; IVS, interventricular septum; LVPW, left ventricular posterior wall. Solid lines and dotted lines represent the median and the quartiles, respectively. P values were calculated by the Mann–Whitney nonparametric test.

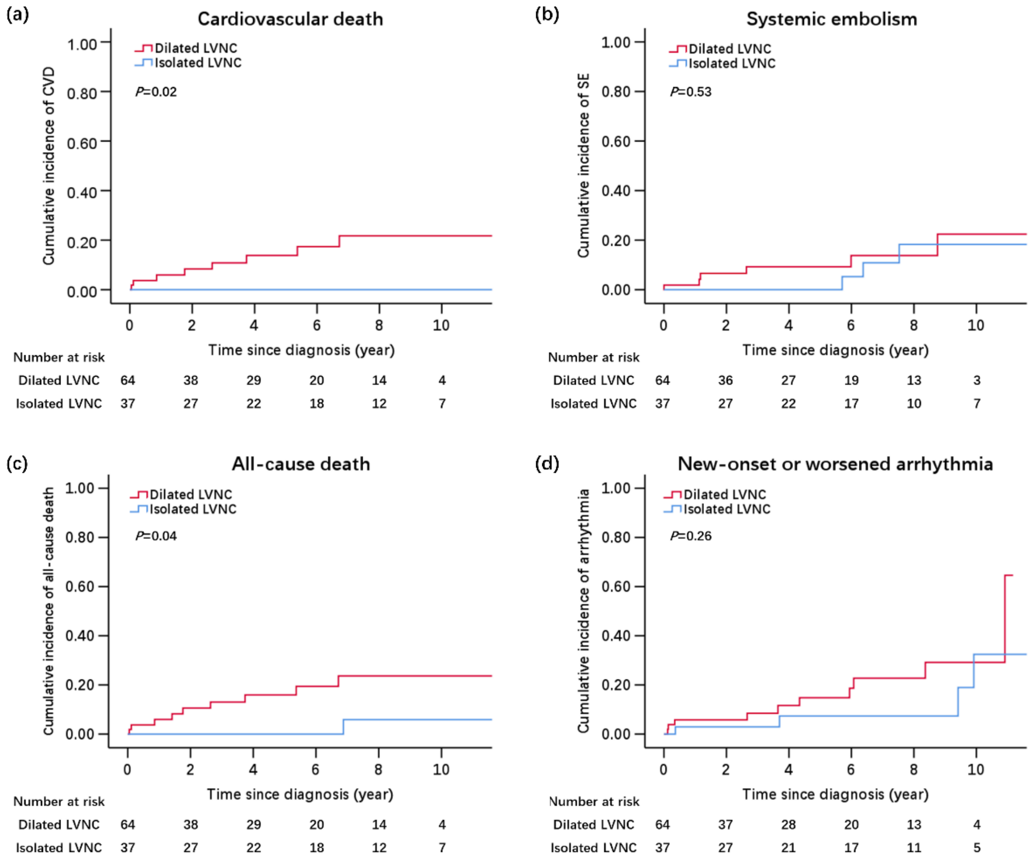

Figure S2. Kaplan-Meier curves for the cumulative incidence of cardiovascular death (a), systemic embolism (b), all-cause death (c) and new onset or worsened arrythmia (d) stratified by isolated LVNC and dilated LVNC.

Abbreviations: LVNC, left ventricular noncompaction; HF, heart failure; HR hazards ratio; CI, confidence interval.

P value was calculated by the log-rank test.

Table S1. Comparison of baseline characteristics between followed-up and dropped-out patients

**Baseline Characteristics**	**Followed-up patients (n=88)**	**Dropped-out patients (n=13)**	***P* Value***
Age of diagnosis, years	46.5±16.3	49.0±18.6	0.62
Age ≥60 y, No. (%)	21 (23.9)	5 (38.5)	0.31
Male, No. (%)	50 (56.8)	7 (53.8)	0.84
Body mass index, kg/m^2	23.1±3.4	24.3±4.7	0.33
NYHA cardiac function class, No. (%)			
I or II	60 (68.2)	6 (46.2)	0.13
III or IV	28 (31.8)	7 (53.8)	
Hypertension, No. (%)	24 (27.3)	5 (8.5)	0.51
Diabetes mellitus, No. (%)	10 (11.4)	2 (15.4)	0.65
Dyslipidemia, No. (%)	28 (31.8)	3 (23.1)	0.75
Coronary heart disease, No. (%)	8 (9.1)	1 (7.7)	>0.99
Cigarette consumption, No. (%)	34 (38.6)	6 (46.2)	0.61
Alcohol intake, No. (%)	34 (38.6)	7 (53.8)	0.30
Abnormal ECG	52 (59.1)	8 (61.5)	>0.99
**Echocardiography features**			
LVEDD, mm	62.0 (54.3–72.5)	58.0 (56.5–72.5)	0.82
LVESD, mm	51.0 (39.0-63.0)	51.0 (44.0-61.0)	0.95
LVEF, %	36.3 (27.0–54.0)	34.0 (21.0–48.5)	0.50
IVS, mm	8.0 (6.9–9.0)	8.0 (7.0–9.0)	0.53
LVPW, mm	8.0 (7.0–9.0)	7.5 (7.0–9.0)	0.94
LA enlargement, No. (%)	64 (72.7)	11 (27.5)	0.51
MV regurgitation, No (%)			
None to mild	65 (73.9)	8 (61.5)	0.34
Moderate to severe	23 (26.1)	5 (38.5)	
**Laboratory examinations**			
Lipids profile, mmol/L			
Total cholesterol	4.07 (3.34–4.77)	3.34 (3.19–4.48)	0.10
Triglycerides	1.17 (0.76–1.76)	0.89 (0.62–1.12)	0.05
HDL-C	0.99 (0.80–1.19)	1.04 (0.66–1.13)	0.71
LDL-C	2.58 (1.95–3.20)	2.31 (1.84–2.58)	0.23
cTnI elevation, No. (%)†	20 (30.8)	3 (27.3)	>0.99
BNP or NT-proBNP elevation, No. (%)§	64 (72.7)	10 (76.9)	>0.99

Abbreviations: LVNC, left ventricular noncompaction; NYHA, New York Heart Association; ECG, electrocardiography; LVEDD, left ventricular end-diastolic diameter; LVESD, left ventricular end-systolic diameter; LVEF, left ventricular ejection fraction; IVS, interventricular septum; LVPW, left

ventricular posterior wall; LA, left atria; MV, mitral valve; HDL-C, high-density lipoprotein cholesterol; LDL-C, low-density lipoprotein cholesterol; cTnI, cardiac troponin I; BNP, brain natriuretic peptide; NT-proBNP, N-terminal pro-brain natriuretic peptide.

Values were given as mean ± SD, number (%), or median (interquartile range).

P values were calculated by Student t test or Mann-Whitney nonparametric test for quantitative variables, and by Chi-square test or Fisher's precise test for qualitative variables, when appropriate.

[†]Elevation in cTnI was defined as baseline cTnI above 99th percentile upper reference limit.

[§]Elevation in BNP or NT-proBNP was defined as BNP >35 ng/L or NT-proBNP >125 ng/L in patients with sinus rhythm, or BNP >105 ng/L or NT-proBNP >365 ng/L in patients with atrial fibrillation.

Table S2. Multivariate analysis of risk factors associated with dilated LVNC

Risk factor	Dilated LVNC (n=64)	Isolated LVNC (n=37)	*P* Value†	Multivariate analysis*	
				OR (95% CI)	*P* value
Male, No. (%)	41 (64.1)	16 (43.2)	0.04	2.21 (0.81, 6.08)	0.12
Age≥60 y, No. (%)	22 (34.4)	4 (10.8)	<0.01	4.19 (1.26, 13.93)	0.02
Body mass index, kg/m^2	22.8±3.5	23.9±3.5	0.19	-	-
Hypertension, No. (%)	16 (25.0)	13 (35.1)	0.28	-	-
Diabetes mellitus, No. (%)	8 (12.5)	4 (10.8)	>0.99	-	-
Coronary heart disease, No. (%)	8 (12.5)	1 (2.7)	0.15	-	-
Dyslipidemia, No. (%)	25 (39.1)	6 (16.2)	0.02	2.64 (0.92, 7.59)	0.07
Cigarette consumption, No. (%)	29 (45.3)	11 (29.7)	0.12	-	-
Alcohol intake, No. (%)	29 (45.3)	12 (32.4)	0.20	1.14 (0.40, 3.23)	0.80

Abbreviations: LVNC, left ventricular noncompaction; OR, odds ratio; CI, confidence interval; NYHA, New York Heart Association.

Values were given as mean ± SD or number (%).

†*P* values were calculated by the Student t-test or Mann-Whitney nonparametric test for quantitative variables, and Chi-square test or Fisher's precise test for qualitative variables, when appropriate.

*OR (95% CI) and *P* values were calculated by using the Logistic regression model.

Table S3. Univariate and multivariate analysis of variables associated with MACE among patients with LVNC*

	Univariate analysis		Multivariate analysis	
	**Crude HR (95% CI)**	***P* value**	**Adjusted HR (95% CI)**	***P* value**
Dilated LVNC	7.62 (2.70, 21.53)	<0.001	4.43 (1.24, 15.81)	0.02
Male	2.48 (1.22, 5.01)	0.01	1.47 (0.60, 3.61)	0.41
Age ≥60 y	2.25 (1.17, 4.37)	0.02	1.39 (0.69, 2.77)	0.36
NYHA functional class III-IV	1.54 (0.80, 2.96)	0.20	0.73 (0.36, 1.47)	0.38
Hypertension	0.67 (0.32, 1.43)	0.30	-	-
Diabetes mellitus	1.26 (0.52, 3.07)	0.61	-	-
Dyslipidemia	1.60 (0.83, 3.09)	0.16	-	-
Coronary heart disease	1.57 (0.61, 4.04)	0.35	-	-
Cigarette consumption	2.05 (1.08, 3.92)	0.03	1.56 (0.69, 3.50)	0.28
Alcohol intake	1.69 (0.89, 3.24)	0.11	-	-
Abnormal ECG	2.91 (1.36, 6.20)	<0.01	1.13 (0.47, 2.13)	0.79
LBBB	2.07 (1.02, 4.20)	0.04	-	-
Atrial fibrillation	1.40 (0.55, 3.60)	0.49	-	-
Ventricular tachycardia	2.09 (0.87, 5.05)	0.10	-	-
LA enlargement	16.94 (2.32,123.67)	<0.01	-	-
Moderate-severe mitral regurgitation	2.02 (1.04, 3.94)	0.04	-	-
cTnI elevation†	2.76 (1.33, 5.71)	<0.01	-	-
BNP/NT-proBNP elevation§	8.77 (2.10, 36.56)	<0.01	2.46 (0.45, 13.43)	0.30

Abbreviations: LVNC, left ventricular noncompaction; NYHA, New York Heart Association; HR, hazards ratio; CI, confidence interval; ECG, electrocardiography; LBBB, left bundle branch block; LA, left atria; cTnI, cardiac troponin I; BNP, brain natriuretic peptide; NT-proBNP, N-terminal pro-brain natriuretic peptide.

HR (95% CI) and *P* values were calculated by using the Cox proportional-hazards regression model.

†Elevation in cTnI was defined as baseline cTnI above 99th percentile upper reference limit.

§Elevation in BNP or NT-proBNP was defined as BNP >35 ng/L or NT-proBNP >125 ng/L in patients with sinus rhythm, or BNP >105 ng/L or NT-proBNP >365 ng/L in patients with atrial fibrillation.

Table S4. Univariate and multivariate analysis of variables associated with heart failure among patients with LVNC

	Univariate analysis		Multivariate analysis	
	Crude HR (95% CI)	*P* value	Adjusted HR (95% CI)	*P* value
Dilated LVNC	11.43 (2.71, 48.26)	< 0.01	7.52 (1.47, 38.52)	0.02
Male	2.44 (1.07, 5.56)	0.03	1.44 (0.50, 4.18)	0.50
Age ≥ 60y	1.82 (0.84, 3.97)	0.13	1.05 (0.46, 2.41)	0.91
NYHA functional class III-IV	1.14 (0.51, 2.54)	0.75	0.50 (0.22, 1.16)	0.11
Abnormal ECG	3.15 (1.27, 7.82)	0.01	1.10 (0.38, 3.13)	0.86
LBBB	1.05 (0.40, 2.78)	0.92	-	-
Atrial fibrillation	2.13 (0.81, 5.63)	0.13	-	-
Ventricular tachycardia	2.51 (0.94, 6.68)	0.07	-	-
LA enlargement	2.29 (1.06, 4.94)	0.04	-	-
Moderate-severe mitral regurgitation	2.02 (1.04, 3.94)	0.04	-	-
cTnI elevation†	2.01 (0.85, 4.73)	0.11	-	-
BNP/NT-proBNP elevation§	12.67 (1.71, 93.24)	0.01	3.52 (0.38, 32.39)	0.27
Hypertension	0.47 (0.18, 1.25)	0.13	-	-
Diabetes mellitus	1.49 (0.55, 4.02)	0.43	-	-
Dyslipidemia	1.11 (0.50, 2.46)	0.81	-	-
Coronary heart disease	1.22 (0.37, 4.07)	0.74	-	-
Cigarette consumption	1.75 (0.82, 3.74)	0.15	1.36 (0.52, 3.58)	0.53
Alcohol intake	1.29 (0.60, 2.79)	0.51	-	-

Abbreviations: LVNC, left ventricular noncompaction; NYHA, New York Heart Association; ECG, electrocardiography; LBBB, left bundle branch block; LA, left atria; cTnI, cardiac troponin I; BNP, brain natriuretic peptide; NT-proBNP, N-terminal pro-brain natriuretic peptide.

HR (95% CI) and *P* value were calculated by using the Cox proportional-hazards regression model.

†Elevation in cTnI was defined as baseline cTnI above 99th percentile upper reference limit.

§Elevation in BNP or NT-proBNP was defined as BNP >35 ng/L or NT-proBNP >125 ng/L in patients with sinus rhythm, or BNP >105 ng/L or NT-proBNP >365 ng/L in patients with atrial fibrillation.
